# Nicotinamide Mononucleotide Adenylyl Transferase 2: A Promising Diagnostic and Therapeutic Target for Colorectal Cancer

**DOI:** 10.1155/2016/1804137

**Published:** 2016-04-27

**Authors:** Chunhui Cui, Jia Qi, Quanwen Deng, Rihong Chen, Duanyang Zhai, Jinlong Yu

**Affiliations:** ^1^Department of General Surgery, Zhujiang Hospital, Southern Medical University, Guangzhou, Guangdong Province, China; ^2^Section of Medicinal Material, Southern Hospital, Southern Medical University, Guangzhou, Guangdong Province, China

## Abstract

Colorectal cancer (CRC) is one of the most common cancers all over the world. It is essential to search for more effective diagnostic and therapeutic methods for CRC. Abnormal nicotinamide adenine dinucleotide (NAD) metabolism has been considered as a characteristic of cancer cells. In this study, nicotinamide mononucleotide adenylyl transferases (NMNATs) as well as p53-mediated cancer signaling pathways were investigated in patients with colorectal cancer. The CRC tissues and adjacent normal tissues were obtained from 95 untreated colorectal cancer patients and were stained for expression of nicotinamide mononucleotide adenylyl transferase 2 (NMNAT2) and p53. The survival rate was analyzed by the Kaplan-Meier method and the log-rank test. The multivariate Cox proportional hazard regression analysis was conducted as well. Our data demonstrated that expression of NMNAT2 and p53 was significantly higher in CRC tissues, while NMNAT2 expression is in correlation with the invasive depth of tumors and TNM stage. Significant positive correlation was found between the expression of NMNAT2 and the expression of p53. However, NMNAT2 expression was not a statistically significant prognostic factor for overall survival. In conclusion, our results indicated that NMNAT2 might participate in tumorigenesis of CRC in a p53-dependent manner and NMNAT2 expression might be a potential therapeutic target for CRC.

## 1. Introduction

The colorectal cancer (CRC) is the third most commonly diagnosed cancer. More than 1.2 million patients are diagnosed with CRC every year, and more than 600,000 people die from the disease [1, 2]. Surgery resection combined with chemotherapy is the principal treatment for CRC. However, the conventional chemotherapeutic drugs, such as 5-fluorouracil, leucovorin, and oxaliplatin, harbor cytotoxicity affecting not only tumor cells but also the normal ones [3]. Therefore, identification of therapeutic targets is needed for developing novel therapy of CRC.

Nicotinamide adenine dinucleotide (NAD) is an oxidoreductase coenzyme that plays a central role in a wide range of biological processes, such as energy metabolism, circadian rhythm, axon survival, calcium mobilization, cell death, and aging [4]. As an oxidoreductase coenzyme, NAD switches between its oxidized form NAD+ and reduced form, and the NAD+/NADH ratio plays an important role in maintaining the intracellular redox equilibrium and controlling the metabolic state of the cell [5]. Changing of NAD+/NADH disturbs the balance of cellular redox and further promotes progression of various diseases [6]. Moreover, NAD+ was also identified as a substrate for the sirtuins (SIRTs) family, which is a class of metabolic regulator and functions as deacetylase proteins [7]. SIRTs could act as metabolic sensors which employ NAD+ as a messenger or cosubstrate to transduce signals for cellular activities [7]. Since NAD is vital not only for energy transduction but also for intracellular signaling pathways, abnormal metabolism of NAD has been considered as a characteristic of tumorigenesis [8]. It is believed that accelerated cell growth and proliferation of tumor cells partially resulted from dysregulation of energy production as well as speeding metabolism [8].

Nicotinamide mononucleotide adenylyl transferases (NMNATs) are rate-limiting enzymes, which catalyze the synthesis of NAD from nicotinamide mononucleotide (NMN). Three NMNAT isoforms have been identified in mammals, including NMNAT1, NMNAT2, and NMNAT3 [4, 9]. Among the three NMNAT isoforms, NMNAT2 is reported to be most sensitive to NAD and can act as a sensor to intracellular metabolic state and high levels of NMNAT2 were detected in the organs with high energy consumption, such as heart, brain, and skeletal muscle [10]. Since cancer cells harbor high demand for energy [11], it is interesting to know if NMNAT2 is upregulated in colorectal carcinoma tissues.

Furthermore, NMNAT2 has been indicated to play an important functional role in p53-mediated cancer suppression process. p53 is a classic tumor suppressor gene that has been also found to play a critical role in regulating metabolism and intracellular signaling pathways [12–14]. SIRTs could utilize NAD+ to catalyze the removal of acetyl groups from p53, resulting in the “silencing” of p53 activity [15]. Therefore, it gives rise to the hypothesis that NAD metabolism and p53 function are intimately linked in CRC.

In this study, we investigate the expression of NMNATs as well as p53-mediated cancer signaling pathways in patients with colorectal cancer. Our data showed that NMNAT2 was significantly upregulated in CRC tissues compared with adjacent normal tissues and was correlated with the invasive depth of tumor and TNM stage. The NMNAT2 level was also correlated with the expression of p53. However, NMNAT2 expression was not a statistically significant prognostic factor for overall survival. In conclusion, our results indicated that NMNAT2 might participate in the tumorigenesis of CRC in a p53-dependent manner and NMNAT2 might be a potential therapeutic target for CRC.

## 2. Material and Methods

### 2.1. Ethics Statement

A total of 95 CRC patients were enrolled in this study. All participating subjects were formally informed for the purpose of this study and every subject involved signed a letter of consent. This study was also reviewed and approved by the Hospital Ethics Committees of Zhujiang Hospital and Nanfang Hospital of Southern Medical University.

### 2.2. Inclusion and Exclusion Criteria

All patients included in this study met the following criteria: (1) diagnosed as primary CRC based on the clinical history, pathological reports, and H&E stained tissue samples; (2) not receiving preoperative radiotherapy, chemotherapy, and other treatments. Patients with a history of other malignant diseases or a metastatic CRC were excluded.

### 2.3. General Information and Samples Collection

These CRC patients were admitted to Zhujiang Hospital and Nanfang Hospital of Southern Medical University between January 2008 and June 2010. The patients consisted of 50 males and 42 females. The age of the patients ranged from 30 to 91 years (median, 61 years). CRC from these patients was classified as well differentiated (*n* = 21), moderately differentiated (*n* = 70), or poorly differentiated (*n* = 4). According to the Cancer TNM staging standard of American Joint Committee, there were 34 patients in stage I, 22 patients in stage II, 22 patients in stage III, and 17 patients in stage IV. The CRC tissues and the adjacent normal tissues, which were located 5 cm away from the tumor margin, were obtained from surgical specimens. All patients had a regular follow-up after surgery. Other clinical data such as the tumor size, morphology type, histology type, depth of invasion, lymph metastasis, and distant metastasis were recorded and analyzed as well.

### 2.4. Immunohistochemical Staining

The tumor and adjacent normal tissues were fixed in formalin and embedded in paraffin and then were processed as 4 *μ*m sections. The sections were deparaffinized and rehydrated by ethanol overnight at 60°C. Heat-induced epitope retrieval was performed by using high-pressure boiler in citrate buffer (pH 6.0) for 3 minutes. After endogenous peroxidase activity had been blocked with 3% hydrogen peroxide, the sections were washed in 0.01 mol/L phosphate-buffered saline solution (PBS, Boster, China) for three times (5 min for each). Then the slides were incubated at 4°C overnight with anti-NMNAT2 antibody (Santa Cruz, CA, USA) or anti-p53 antibody (ZSGB, China). Specific reactions between antibodies and target proteins were visualized by probing the slides with HRP-conjugated secondary antibodies and 3,3′-diaminobenzidine (DAB, Gene Tech, CA). The counterstaining was conducted with Mayer's hematoxylin as well. The slides incubated with PBS without primary antibodies were included as negative controls. All slides were observed with an Olympus microscope and images were recorded by digital camera.

The stained slides were assessed independently by two clinical pathologists without prior knowledge of pathological parameters under a light microscope. Only cytoplasmic reactivity for NMNAT2 or nuclear reactivity for p53 was considered as positive staining. The staining intensity and the percentage of the positive cells were evaluated. The staining degree of protein was scored as 0 (negative), 1 (weak), 2 (moderate), and 3 (strong). The mean percentage of positive cells was quantified by calculating at least five different fields with 200x magnification and classified into five categories: 0, <5%; 1, 5%–25%; 2, 25%–50%; 3, 50%–75%; 4, >75%. The final score was calculated as the intensity score plus the quantity score. Staining for the proteins was considered to be positive when the final score was higher than or equal to 3.

### 2.5. Follow-Up Observations

All the cases had a regular follow-up after surgery. Besides the conventional postsurgery examinations and treatments, patients were interviewed by telephone or e-mails with an interval of 3 months and 6 months for the first 2 years and after 2 years, respectively. The total follow-up period was 5 years. The terminal event of follow-up observations was defined as death due to any causes.

### 2.6. Statistical Analysis

All data were presented as count or percentage for discrete variables. Paired *t*-test was performed to analyze the expression of NMNAT2 and p53 in tumor tissues and adjacent normal tissues. The pathological parameters among groups were compared by the chi-square test or the Fisher exact test. The association of the expression level between NMNAT2 and p53 was assessed by Spearman's nonparametric correlation. The survival rate was analyzed by the Kaplan-Meier method and the log-rank test. The Cox proportional hazard regression model was conducted in multivariate analysis to identify risk factors for overall survival rate (OS). *P* < 0.05 was considered as statistically significant. The statistical analysis was performed by using SPSS software version 20.0 (SPSS Inc., Chicago, IL, USA).

## 3. Results

### 3.1. Expression Level of NMNAT2 and p53 in CRC

**Figure 1:**
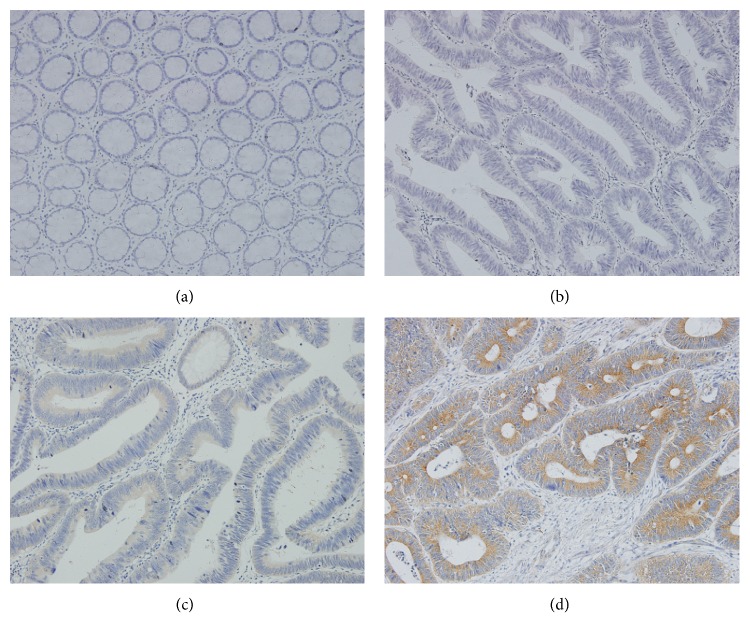
NMNAT2 immunohistochemical staining for colorectal cancer peritumoral tissues (×200). (a) Negative (intensity score 0, percentage score 0) and (b) negative (intensity score 1, percentage score 1) and (c) positive (intensity score 2, percentage score 3) and (d) positive (intensity score 3, percentage score 3).

**Figure 2:**
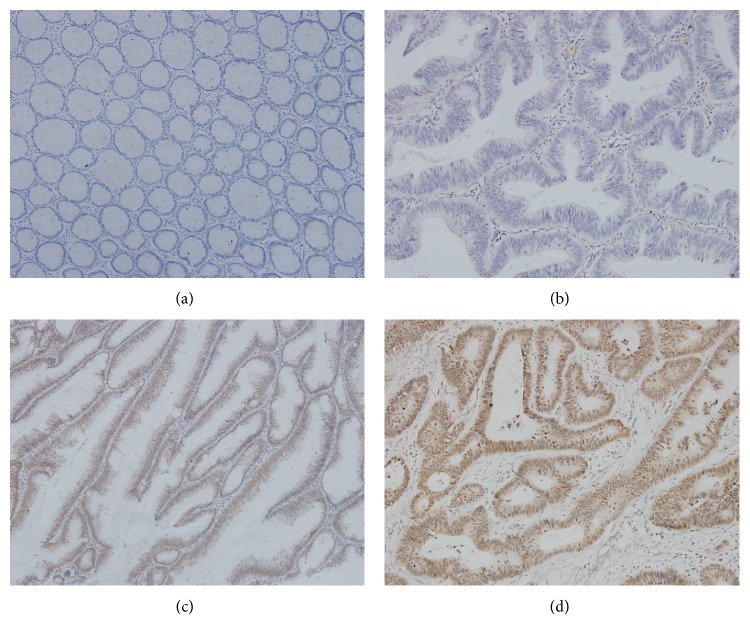
p53 immunohistochemical stain for colorectal cancer peritumoral tissues (×200). (a) Negative (intensity score 0, percentage score 0) and (b) negative (intensity score 0, percentage score 0) and (c) positive (intensity score 2, percentage score 3) and (d) positive (intensity score 3, percentage score 3).

NMNAT2 protein expression was localized primarily in cytoplasm and the diffusely cytoplasmic brown-staining was observed in positive tissues as shown in Figure 1. The specificity of the anti-NMNAT2 antibody was confirmed by western blot measuring different pairs of tumor-peritumoral tissue samples of CRC (see Figure S1 in Supplementary Material available online at http://dx.doi.org/10.1155/2016/1804137). The NMNAT2 expression could be detected in 78 of 95 (82.1%) cases among CRC tissues compared to 34 of 95 (35.7%) for adjacent normal tissues, which suggested NMNAT2 was significantly upregulated in CRC (Table 1). On the other hand, p53 protein was mainly presented in nuclear of cancer cells (Figure 2). Our results showed that the protein expression of p53 in CRC tissues (60 of 95, 63.1%) was significantly more than adjacent normal tissues (16 of 95, 16.8%) (*P* < 0.01).

### 3.2. The Expressive Relationship between NMNAT2 and p53

According to recent reports, the* NMNAT2* plays a key role in p53-mediated cancer signaling pathways. Further analysis demonstrated that NMNAT2-positive tumor tissues showed significant superiority over the adjacent normal tissues with respect to the p53 positive samples (*P* < 0.05). Moreover, for the tumor tissues which was p53 negative, a similar NMNAT2-positive pattern was observed between tumor tissue and adjacent tissues (*P* > 0.05). Furthermore, positive correlation was found between the expression of NMNAT2 and p53 (*r* = 0.428, *P* < 0.05, Table 2).

### 3.3. Clinical Parameters Correlated with NMNAT2

The demographic date and various pathologic parameters of 95 patients were investigated as well. Correlation between NMNAT2, p53 protein expression, and various parameters was shown in Table 3. No significant differences in age, gender, tumor size, morphology type, histological type, degree of lymphatic metastasis, and distant metastasis were observed between the positive group and negative group (*P* > 0.05). However, the expression of NMNAT2 in CRC tissues was in correlation with the invasive depth of tumor and TNM stage (*P* < 0.05). Compared with patients within TNM stage I/II, NMNAT2-positive tumor tissues were more observed in TNM stage III/IV patients (Figure S2).

### 3.4. NMNAT2 Expression and Survival Analysis

**Figure 3:**
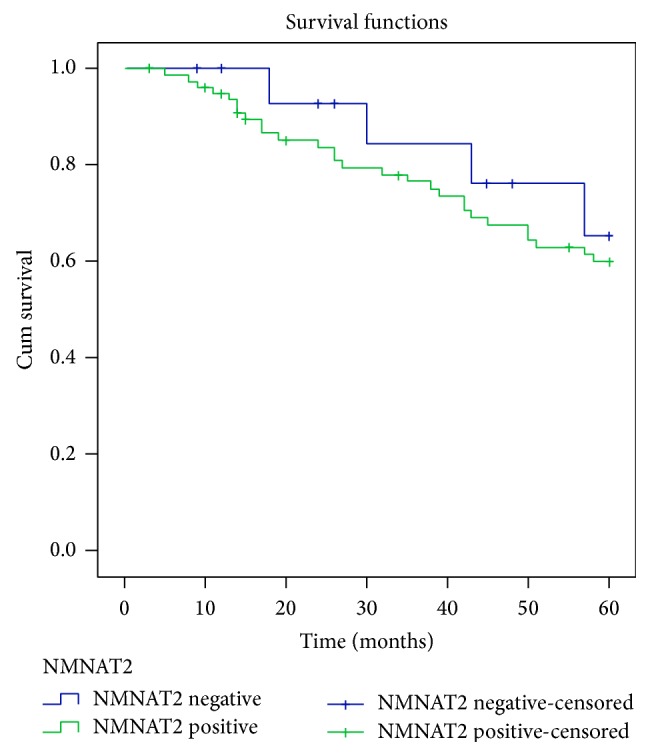
Survival analysis comparative curves between negative NMNAT2 expression and positive NMNAT2 expression in CRC patients.

The 5-year survival rate was 76.5% in the NMNAT2-negative group and 64.1% in the NMNAT2-positive group (*P* = 0.531, Figure 3). The log-rank test indicated that the 5-year survival rate was not associated with the NMNAT2 expression. According to the multivariate analysis, the prognostic factors for survival were age (*P* = 0.002) and TNM stages (*P* = 0.01). However, NMNAT2 level was not a statistically significant prognostic factor (*P* = 0.122) (Table 4).

## 4. Discussion

As “Warburg effect” suggests that tumor cells consume glucose at higher rate than normal cells and secrete most of the glucose-derived carbon as lactate instead of oxidizing it completely due to rapid proliferation, metabolic reprogramming has become a biological feature of cancer [10, 11]. Dysregulation of cellular energy metabolism can lead to various types of disorders, including coronary heart disease, diabetes, obesity, and cancers [12–14]. As an oxidoreductase coenzyme, NAD is involved in multiple intracellular signaling pathways due to its ability to catalyze cellular metabolic activity [16, 17]. Maintaining intracellular NAD confers survival advantage in various diseases and can be reflected by NAD [18–20].

Compared with ubiquitously expressed NMNAT1 and NMNAT3, NMNAT2 mRNA was reported to be mainly expressed in high energy consumption tissues such as brain, heart, skeletal muscle, and some tumor tissues [7, 8]. Previous studies mainly focused on NMNAT2's role in neurodegenerative diseases (e.g., Alzheimer's disease), which demonstrated that NMNAT2 could prevent neuron and axon from apoptosis [21–23]. Recent studies also indicated that the function of NMNAT2 is partially overlapped with SIRT3, which improves mitochondrial functions, and is related to energy metabolism in human non-small cell lung carcinoma (NSCLC) cell lines A549. Reports also suggested that the binding of SIRT3 with NMNAT2 is a novel regulator of cell proliferation and apoptosis in NSCLC cell lines [24]. Therefore, NMNAT2 may play a potential role in promoting proliferation and inhibiting apoptosis in CRC cells as well.

Here, our data showed that NMNAT2 was expressed to be higher in CRC tissues than normal tissues. In addition, we found that the expression of NMNAT2 protein in tumor tissues had a significant correlation with the invasive depth of tumor and TNM stages according to the statistics (*P* < 0.05). This suggested that NMNAT2 may make a significant contribution to tumorigenesis and development of CRC by maintaining intracellular NAD to fuel the rapid cell growth and proliferation.

Since p53 and NAD are both proved to influence the energy metabolism in cancer cells and the acetylation of p53 is regulated by NAD-dependent SIRT1 deacetylase [9, 25], the NAD metabolism may be potentially linked with p53 function in CRC tissues. As a tumor suppressor, p53 is frequently mutated in various neoplastic types during tumorigenesis [26]. The mutation of p53 is a critical step for the progression of colorectum from adenoma to adenocarcinoma [27]. In our study, the expression of mutated p53 in CRC tissues was observed as well. Our results demonstrated that p53 expression was significantly upregulated in CRC tissues and correlated with invasive depth of tumor, TNM stage, and lymphatic metastasis (*P* < 0.05), which was consistent with the earlier studies [28, 29]. It is notable that positive correlation was found between the expression of NMNAT2 and p53 (*r* = 0.428, *P* < 0.05). The NMNAT2-positive tumor tissues showed significant superiority over adjacent normal ones with respect to the p53 positive samples (*P* < 0.05). However, in p53 negative samples, a similar NMNAT2-positive rate was observed between tumor tissues and adjacent normal tissues (*P* > 0.05).

Our data suggested that NMNAT2 is likely to be a key linker between NAD and p53, and its expression is correlated with p53 in CRC, which is consistent with previous speculations that NMNAT2 is a downstream target of p53 and NMNAT2 could be induced in a p53-independent manner in human CRC cell line HCT116 [30]. Moreover, the serum anti-p53 antibody has been proposed as a promising candidate biomarker for early detection of CRC, and immunohistochemical staining of p53 has been proposed as a routine test for CRC [31]. Therefore, it may imply that NMNAT2 expression in CRC tissues could be a diagnostic predictor for CRC patients.

On the other hand, the enzymes involved in NAD dysfunctional metabolism are attractive targets for developing targeted therapy for diseases such as obesity, diabetes, neurodegeneration, and cancer [32]. For example, PARP inhibitors are being investigated for chemoprevention, radiosensitization, and anticancer agents [33]. Moreover, the downregulation or reduced activity of NAD-dependent 15-hydroxyprostaglandin dehydrogenase (15-PGDH) is closely related to the development of gastroenteric tumor, which suggests 15-PGDH as a novel target for gastroenteric tumor screening or therapy [34]. Tiazofurin was shown to demonstrate limited cytotoxicity for CRC cells compared with leukemic cell lines. Both in vitro and in vivo studies showed that CRC cell lines require higher dose of Tiazofurin to exhibit antitumor activity [35]. This may imply that NMNAT2 could also be a promising therapeutic target or adjuvant target. However, this speculation still needs further investigation.

On the other hand, our data showed that the 5-year survival rate of CRC patients is not associated with NMNAT2 expression. This might be due to multiple regulars and interference factors for NMNAT2 and survival state, respectively. So further, more studies for specific mechanism are still needed. According to the multivariate analysis, the prognostic factors for survival are age (*P* = 0.002) and TNM stage (*P* = 0.010), which is in line with other studies. In conclusion, our results indicated that NMNAT2 protein is significantly upregulated in CRC tissues and increased expression is in step with the p53 during tumorigenesis. NMNAT2 may be a novel opportunity for diagnosis and treatment of CRC.

## Supplementary Material

Figure S1. The expression of NMNAT2 protein in different pairs of tumor-peritumorial tissue samples of CRC was measured by western blot.
Figure S2. NMNAT2 Immunohistochemical staining in CRC tissues with different TNM stage (×200).

## Figures and Tables

**Table 1:** Expression level of NMNAT2 and p53 in CRC and adjacent normal tissues.

Protein type	IHC stain	Normal tissues	Tumor tissues	Paired-samples *t*-test *P* value
NMNAT2	Positive (*n*)	34	78	*P* = 0.001
	Negative (*n*)	61	17	

p53	Positive (*n*)	16	60	*P* = 0.000
	Negative (*n*)	79	35	

**Table 2:** Correlation of positive expression of NMNAT2 and p53 in colorectal cancer.

	p53 (−)	p53 (+)
NMNAT2 (−)	14	3
NMNAT2 (+)	21	57

	35	60

*r* = 0.440, *P* < 0.001.

**Table 3:** Comparison of positive expression of NMNAT2 and p53 between different clinical pathological characteristic groups of colorectal cancer.

Parameters	Patients (*n*)	NMNAT2			p53		
		Negative	Positive	*P* value	Negative	Positive	*P* value
Gender							
Male	50	9 (18.0%)	41 (82.0%)	*P* = 0.977	18 (36.0%)	32 (64.0%)	*P* = 0.858
Female	45	8 (17.8%)	37 (82.2%)		17 (37.8%)	28 (62.2%)	
Age							
≥65	51	6 (11.8%)	45 (88.2%)	*P* = 0.093	18 (35.3%)	33 (64.7%)	*P* = 0.736
<65	44	11 (25.0%)	33 (75.0%)		17 (38.6%)	27 (61.4%)	
Morphology							
Ulcerative type	65	12 (18.5%)	53 (81.5%)	*P* = 0.832	26 (40.0%)	39 (60.0%)	*P* = 0.348
Polypoid type	30	5 (16.7%)	25 (83.3%)		9 (30.0%)	21 (70.0%)	
Histological type							
Adenocarcinoma	77	13 (16.9%)	64 (83.1%)	*P* = 0.733	29 (37.7%)	48 (62.3%)	*P* = 0.732
Mucous carcinoma	18	4 (22.2%)	14 (77.8%)		6 (33.3%)	12 (66.7%)	
Differentiation							
Well	21	3 (14.3%)	18 (85.7%)	*P* = 0.756	10 (47.6%)	11 (52.4%)	*P* = 0.246
Middle and poor	74	14 (18.9%)	60 (81.1%)		25 (33.8%)	49 (66.2%)	
Average diameter							
≥3	52	8 (15.4%)	44 (84.6%)	*P* = 0.483	17 (32.7%)	35 (67.3%)	*P* = 0.356
<3	43	9 (20.9%)	34 (79.1%)		18 (41.9%)	25 (58.1%)	
Depth of invasion							
T1 + T2	44	13 (29.5%)	31 (70.5%)	*P* = 0.006	25 (56.8%)	19 (43.2%)	*P* < 0.001
T3 + T4	51	4 (7.8%)	47 (92.2%)		10 (19.6%)	41 (80.4%)	
Lymph metastasis							
N0	60	14 (23.3%)	46 (76.7%)	*P* = 0.070	28 (46.7%)	32 (53.3%)	*P* = 0.009
N1 + N2	35	3 (8.6%)	32 (91.4%)		7 (20.0%)	28 (80.0%)	
TNM stage							
I + II	56	14 (25.0%)	42 (75.0%)	*P* = 0.030	26 (46.4%)	30 (53.6%)	*P* = 0.020
III + IV	39	3 (7.7%)	36 (92.3%)		9 (23.1%)	30 (76.9%)	

**Table 4:** Univariate and multivariate Cox regression analyses of prognostic factors for 5-year survival.

Parameters	Univariate	HR (95% CI)	Multivariate
Gender			
Male	*P* = 0.731		*P* = 0.105
Female			
Age			
≥65	*P* = 0.004		*P* = 0.729
<65			
Morphology			
Ulcerative type	*P* < 0.001	3.105 (1.165–8.273)	*P* = 0.023
Polypoid type			
Histological type			
Adenocarcinoma	*P* = 0.195		*P* = 0.201
Mucous carcinoma			
Differentiation			
Well	*P* = 0.018		*P* = 0.123
Middle and poor			
Average diameter			
≥3	*P* < 0.001	2.993 (1.480–6.054)	*P* = 0.002
<3			
Depth of invasion			
T1 + T2	*P* = 0.002		
T3 + T4			
Lymph metastasis			
N0	*P* < 0.001		
N1 + N2			
TNM stage			
I + II	*P* < 0.001	4.508 (2.303–8.822)	*P* < 0.001
III + IV			
NMNAT2 expression			
Negative	*P* = 0.535		*P* = 0.369
Positive			
p53 expression			
Negative	*P* = 0.806		*P* = 0.767
Positive			
