# Supplementary material for: Nicotinamide Mononucleotide Adenylyl Transferase 2: A Promising Diagnostic and Therapeutic Target for Colorectal Cancer
Source: Biomed Res Int. 2016 Apr 27;2016:1804137. doi: 10.1155/2016/1804137 (PMC4863092; doi:10.1155/2016/1804137)
Supplement: Supplementary file 1 — Figure S1. The expression of NMNAT2 protein in different pairs of tumor-peritumorial tissue samples of CRC was measured by western blot. Figure S2. NMNAT2 Immunohistochemical staining in CRC tissues with different TNM stage (×200). [file 1804137.f1.pdf]

Fig. S1 The expression of NMNAT2 protein in different pairs of tumor- peritumorial tissue samples of CRC was measured by western blot.

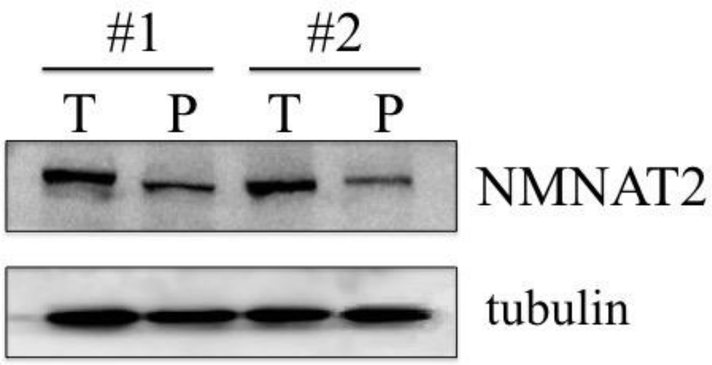

T represents tumor tissue, and P represents corresponding peritumorial tissue.

Fig. S2. NMNAT2 Immunohistochemical staining in CRC tissues with different TNM stage (×200)

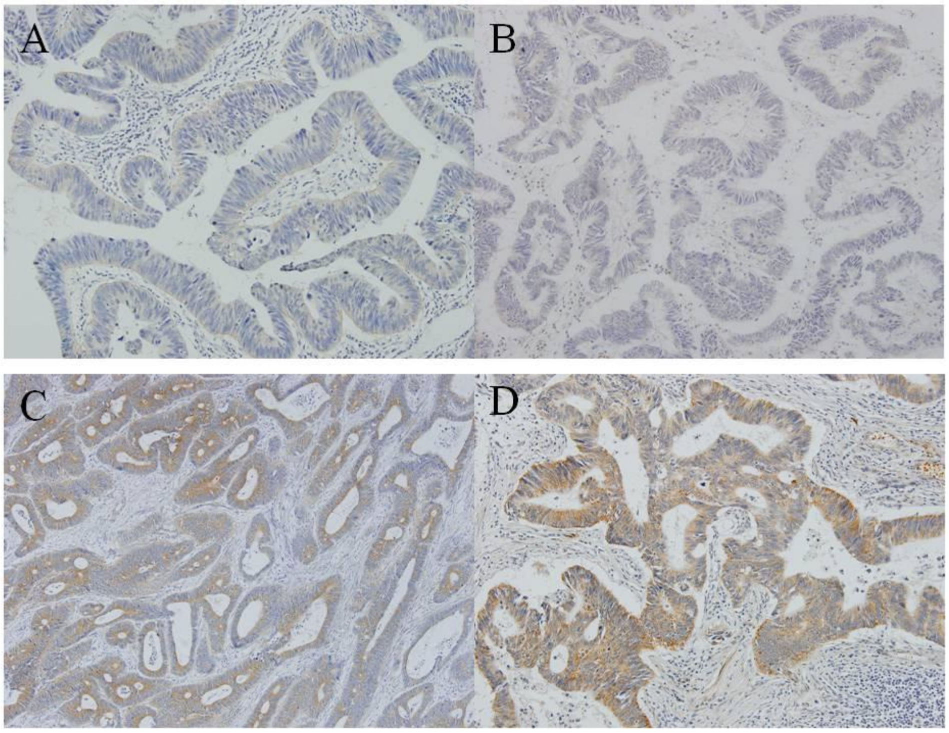

(A) negative in TNM stage I (intensity score 0, percentage score 0)and(B) negative in TNM stage II (intensity score 1, percentage score 1)and(C)positive in TNM stage III (intensity score 2, percentage score 3)(D)positive in TNM stage IV (intensity score 3, percentage score 3)
